# Thymine DNA glycosylase exhibits negligible affinity for nucleobases that it removes from DNA

**DOI:** 10.1093/nar/gkv890

**Published:** 2015-09-10

**Authors:** Shuja S. Malik, Christopher T. Coey, Kristen M. Varney, Edwin Pozharski, Alexander C. Drohat

**Affiliations:** 1 Department of Biochemistry and Molecular Biology, University of Maryland School of Medicine, Baltimore, MD 21201, USA; 2 University of Maryland Marlene and Stewart Greenebaum Cancer Center, Baltimore, MD 21201, USA; 3 Center for Biomolecular Therapeutics, Institute for Bioscience and Biotechnology Research, Rockville, MD 20850, USA

## Abstract

Thymine DNA Glycosylase (TDG) performs essential functions in maintaining genetic integrity and epigenetic regulation. Initiating base excision repair, TDG removes thymine from mutagenic G**·**T mispairs caused by 5-methylcytosine (mC) deamination and other lesions including uracil (U) and 5-hydroxymethyluracil (hmU). In DNA demethylation, TDG excises 5-formylcytosine (fC) and 5-carboxylcytosine (caC), which are generated from mC by Tet (ten–eleven translocation) enzymes. Using improved crystallization conditions, we solved high-resolution (up to 1.45 Å) structures of TDG enzyme–product complexes generated from substrates including G·U, G·T, G·hmU, G·fC and G·caC. The structures reveal many new features, including key water-mediated enzyme–substrate interactions. Together with nuclear magnetic resonance experiments, the structures demonstrate that TDG releases the excised base from its tight product complex with abasic DNA, contrary to previous reports. Moreover, DNA-free TDG exhibits no significant binding to free nucleobases (U, T, hmU), indicating a *K*_d_ >> 10 mM. The structures reveal a solvent-filled channel to the active site, which might facilitate dissociation of the excised base and enable caC excision, which involves solvent-mediated acid catalysis. Dissociation of the excised base allows TDG to bind the beta rather than the alpha anomer of the abasic sugar, which might stabilize the enzyme–product complex.

## INTRODUCTION

Thymine DNA glycosylase (TDG) is a base excision repair (BER) enzyme that acts on derivatives of 5-methylcytosine (mC) arising from deamination or oxidation. TDG excises thymine from G·T mispairs, an activity that is needed to protect against C→T transition mutations caused by deamination of mC to T (1,2). It also plays an essential role in active DNA demethylation, which likely accounts for findings that its depletion in mice causes embryonic lethality (3,4). One established pathway for active DNA demethylation involves TDG excision of 5-formylcytosine or 5-carboxylcytosine (5,6), oxidation derivatives of mC generated by TET (ten–eleven translocation) enzymes (6–10). In addition to these key substrates, TDG removes many other bases (*in vitro*), including uracil, 5-halogenated uracils (5FU, 5ClU, 5BrU, 5IU) and 5-hydroxymethyl-U (hmU) (11,12). Human TDG is comprised of a catalytic domain of about 190 residues, flanked by two regions (about 110 residues each) that are disordered but important for certain functions, interactions with other proteins and regulation by post-translational modifications such as acetylation, phosphorylation and SUMO conjugation (13–18).

Crystal structures of TDG have revealed many details regarding its specificity and mechanism of catalysis and how SUMO proteins bind and alter its structure. The first reported structures were of SUMO-conjugated TDG (residues 117–332) (19,20); which defined its SUMO-interacting motif (SIM) and suggested a mechanism to explain findings that SUMO conjugation weakens its affinity for abasic DNA (18,21). The first structure of DNA-bound TDG was of the catalytic domain (TDG^cat^; residues 111–308), with a tetrahydrofuran (THF) abasic site analog flipped into its active site (22). This structure of the enzyme–product (E·P) complex revealed contacts with the mismatched guanine that may confer some specificity for G·T mispairs, and contacts with a flanking (3′) guanine that may impart specificity for a CpG dinucleotide context (22). However, because the E·P complex was prepared using the THF analog, the structure did not inform potential TDG interactions with the C1’ hydroxyl of the natural abasic site, nor did it reveal whether the excised base is retained in the product complex. Structures were subsequently reported for TDG^cat^ bound to a G·U mispair (23) and to a G·caC pair (24), with non-cleavable (2′-fluoroarabino) deoxynucleotide analogs of U and caC flipped into the active site. These structures reveal contacts with the flipped base in the productive enzyme–substrate (E·S) complex.

**Figure 1.**
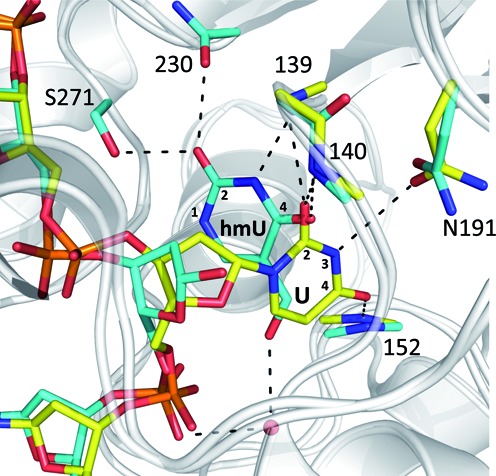
Alignment of two previous structures for TDG^cat^. A structure of the enzyme–product (E·P) complex for TDG^cat^ processing of a G·hmU mispair is shown with DNA and interacting enzyme moieties colored in cyan (PDB ID: 4FNC) (25). Aligned with this is a structure of the enzyme–substrate (E·S) complex for a G·U^F^ mispair, where U^F^ is a dU analog that flips but is not cleaved by TDG, with DNA and enzyme moieties colored yellow (PDB ID: 3UFJ). For the reported product complex, the putative excised hmU base is markedly displaced from its expected position prior to C-N bond cleavage, forming different contacts with TDG compared to those expected prior to bond cleavage (as indicated by U contacts in the E·S complex). Labels for side chains include the residue type; those for backbone groups include residue number only. Relevant positions of hmU and U are indicated.

Reported more recently was the structure of an E·P complex resulting from TDG^cat^ action on a G·hmU mispair in DNA (25), which is remarkable in two respects. First, the excised hmU is reportedly trapped in the active site, despite its relatively low concentration, and, second, hmU is displaced from the abasic sugar, suggesting it moves to a new active-site location after bond cleavage (Figure 1). Given the conditions for the crystallization sample (0.2 mM G·hmU substrate, 0.35 mM TDG^cat^) (25) and the activity of TDG for hmU excision (*k*_max_ = 2 min^-1^) (11), the substrate was fully converted to products prior to crystallization, giving a 0.2 mM concentration of hmU and abasic DNA. If hmU remains bound in a ternary product complex, as reported, it would suggest that TDG possesses unusually high affinity for the excised base, given the relatively low hmU concentration in the crystallization sample and that crystals were cryoprotected by soaking in a solution that lacked hmU (25). Notably, for the bacterial MUG (mismatch-specific uracil glycosylase) enzymes, which are the closest homologs to TDG (32% sequence identity with *Escherichia coli* MUG) (22,26), the excised uracil is not observed in a structure (2.35 Å) of the E·P complex derived from MUG and a G·U substrate (27). While uracil DNA glycosylase (UNG) traps the excised uracil in the E·P complex (28,29), it binds uracil with unusually high affinity, due in part to a strong hydrogen bond (30,31), which is not observed for TDG. Structures of other glycosylases that feature the excised base in an E·P complex, including SMUG1 (single-strand selective monofunctional uracil-DNA glycosylase) (32) and TAG (3-methyladenine DNA glycosylase I) (33), were generated by growing or soaking crystals in the presence of a large molar excess of the nucleobase.

The reported structure of the TDG^cat^ G·hmU product complex also suggests that, in a discrete step after glycosidic bond cleavage, the hmU base rotates and moves 5 Å relative to its expected pre-cleavage position, forming new active-site contacts at O2, N3 and O4 (25) (Figure 1). Although a structure of the E·S complex for G·hmU has not been reported, the pre-cleavage position of hmU likely resembles that of uracil in the E·S complex for a G·U mispair (Figure 1) (23,24). For UNG, the position of uracil and its active-site contacts are largely conserved in the pre- and post-excision (E·S and E·P) complexes (29,34). For TAG, contacts with 3-methyladenine (m^3^A) are the same in the binary complex (TAG·m^3^A) and a ternary complex that also includes abasic DNA (33,35,36). Similar findings are reported for MutY (37,38) and 8-oxoguanine DNA glycosylase (39,40). Thus, to our knowledge, the dramatic post-cleavage relocation of the hmU base proposed for TDG (25) would be unprecedented for a DNA glycosylase.

However, it is important to consider this possibility because a mechanism whereby the enzyme separates the excised base from the abasic sugar after C-N bond cleavage could potentially contribute to catalysis. DNA glycosylase reactions investigated to date follow a stepwise mechanism, where cleavage of the *N*-glycosidic bond yields a discrete though short-lived oxacarbenium ion intermediate, followed by nucleophile addition (41–45). For some glycosylases the first step is reversible and the C-N bond breaks and reforms repeatedly prior to irreversible nucleophile addition (41). As such, sequestration of the leaving group (LG) could potentially suppress reformation of the C-N bond and favor nucleophile addition, depending on the rate of LG displacement relative to that of nucleophile addition. Likewise, release of the excised base could also favor nucleophile addition, again depending on the rate. Thus, it is important to establish whether TDG retains the excised base in its product complex, and if the base is displaced away from the abasic sugar, for hmU and other substrates.

To address this problem, we developed improved crystallization conditions for TDG and solved high-resolution (up to 1.45 Å) structures of product complexes generated from substrates including G·hmU, G·T, G·U, G·fC and G·caC. The new crystallization conditions yield structures of DNA-bound TDG that are of much higher resolution than those previously reported (up to 2.49 Å), revealing details that were not observed in previous structures. These improved crystallization conditions are expected to facilitate future structural studies of TDG. We also studied the product complex in aqueous solution, using nuclear magnetic resonance (NMR) spectroscopy and investigated the affinity of TDG for isolated nucleobases (U, T, hmU). The results define the constituents and nature of the product complex for TDG^cat^, revealing previously unobserved features and informing its mechanism of catalysis. We propose a model whereby release of the excised base could tighten TDG binding to abasic DNA.

## MATERIALS AND METHODS

### Materials

Human TDG (full length), and its catalytic domain (TDG^cat^; residues 111–308), were expressed in *E. coli* and purified essentially as described (22,46). The enzymes were >99% pure as judged by sodium dodecyl sulphate-polyacrylamide gel electrophoresis (Coomassie stained gel) and concentrations were determined by absorbance (47,48). Uniformly ^15^N-labeled TDG^cat^ was produced by expression in MOPS minimal media with 99% [^15^N]-NH_4_Cl (1 g/l) (C.I.L.) (49,50). Briefly, transformed BL21 (DE3) cells (Novagen) were grown overnight on an LB plate (37°C), then several colonies were used to inoculate 0.2 l of LB medium, and grown (37°C) to OD_600_ = 0.6. Cells were harvested, suspended in 2 l of MOPS minimal media, and grown to OD_600_ = 0.7. The temperature was reduced to 15°C, expression was induced with IPTG (0.4 mM) overnight (∼16 h).

Oligodeoxynucleotides (ODNs) were obtained (trityl-off) from the Keck Foundation Biotechnology Resource Laboratory, Yale University, or IDT. ODNs were purified by reverse phase HPLC using an XBridge OST C18 column (Waters Corp.), with mobile phases of 0.1 M TEAA pH 7.0, 5% acetonitrile (A) and 0.1 M TEAA pH 7 15% acetonitrile (B), a flow rate of 5 ml/min, and a gradient of 25 to 55% B over 16 min. Purified ODNs were exchanged into 0.02 M Tris–HCl pH 7.5, 0.04 M NaCl, and their concentration was determined by absorbance (46). ODNs containing the 2′-fluoroarabino analogs of deoxyuridine or deoxythymidine, referred to as U^F^ and T^F^, respectively, were synthesized at Yale using phosphoramidites obtained from Glen Research (U^F^) or Link Technologies (T^F^) (51). TDG binds productively to U^F^ and to T^F^ but these analogs are fully resistant to glycosidic bond cleavage (48,51–53), because the subtle, single-atom substitution destabilizes the chemical transition-state.

DNA for crystal structures included a 28mer strand, 5′-AGCTGTCCATCGCTCAxGTACAGAGCTG, where x is the base excised by TDG, and a complement, 5′-CAGCTCTGTACGTGAGCGATGGACAGCT, that pairs G with the abasic site. The target base (x) is in a CpG context (underlined), consistent with TDG specificity (46,54). This construct was also used for electrophoretic mobility shift assays (EMSAs), with a 3′ 6-FAM on the complementary strand. DNA for NMR included a 23mer, 5′-CCACTGCTCAxGTACAGAGCTGT, where x is the abasic site resulting from TDG^cat^ activity, and a complement, 5′- CAGCTCTGTACGTGAGCAGTGGA-3′, which pairs G with the abasic site, giving a 22-bp duplex with 3′ A or T overhangs and with the AP site in a CpG context (22).

### X-ray crystallography

Samples used for crystallization contained 0.35 mM TDG_cat_ and 0.42 mM DNA in a buffer of 5 mM Tris–HCl pH 7.5, 0.13 M NaCl, 0.2 mM dithiothreitol, 0.2 mM ethylenediaminetetraacetic acid. E·P complexes were produced by incubating TDG^cat^ with DNA substrate for a sufficient time to ensure full conversion to product, confirmed by HPLC (11). Crystals were grown at room temperature (∼22°C) by sitting drop vapor diffusion, using 1 μl of the TDG^cat^-DNA sample and 1 or 2 µl of mother liquor, which was 30% (w/v) PEG 4000, 0.2 M ammonium acetate, 0.1 M sodium acetate, pH 6.0. Crystals typically appeared within in a few days. Crystals were cryo-protected using mother liquor supplemented with 18% ethylene glycol (and other components as noted), and flash cooled in liquid nitrogen.

X-ray diffraction data were collected at the Stanford Synchrotron Radiation Lightsource (SSRL; beamlines 7–1 and 12–2). The images were processed and scaled using Mosflm (55) and Aimless (56) from the CCP4 program suite (57). Alternatively, data processing was performed using the autoxds script developed by Ana Gonzalez and Yingssu Tsai (58) (http://smb.slac.stanford.edu/facilities/software/xds). Whenever possible, we took advantage of the shape of these crystals (long thin blades) to collect multiple datasets that could later be merged to increase resolution. Resolution cutoff was determined based on CC1/2 values (59). The structure of the product complex resulting from the G-hmU substrate (PDB ID: 4XEG) was solved by molecular replacement using Phaser (60) and a previous structure of DNA-bound TDG^cat^ as the search model (PDB ID: 4FNC). All other structures reported were determined by molecular replacement using our new structure (PDB ID: 4XEG) as the search model. Refinement was performed using BUSTER-TNT (61) or REFMAC5 (62), and model building was performed using Coot (63). Specifically, every structural model was initially refined with REFMAC5 (restrained to ideal geometry and using isotropic B factors). Possible TLS groups were identified using the TLSMD server (64,65), and *R*_free_ values were used to select the best model. The structure of an E·P complex derived from a G·U substrate (PDB ID: 4Z47) was deemed amenable to anisotropic B factor refinement, given that it produced similar reduction in both *R* and *R*_free_ and that the sphericity distribution was consistent with expectations as predicted by the PARVATI server (66). Final models were refined using both REFMAC5 and BUSTER-TNT, and refinement protocol chosen based on lower *R*_free_ values. While BUSTER-TNT refinement produced models with systematically better geometry, electron density maps did not show any significant differences that might have altered conclusions reached in this work. The structural figures were made with PyMOL (http://www.pymol.org).

### NMR spectroscopy

^15^N-TROSY and ^15^N-HSQC experiments were collected on a 950 MHz Bruker Avance III NMR spectrometer, and the data were processed and analyzed using NMRPipe and NMRDraw (67). The sample conditions are provided in the relevant figure legends. For samples of TDG^cat^ bound to DNA containing a pre-existing abasic (AP) site, the abasic site was generated by treating DNA containing a G·U mispair with a catalytic amount of UNG (1:500 molar ratio UNG:DNA). For all other E·P complexes, samples were generated by adding TDG^cat^ to substrate DNA (G·U, G·T or G·hmU) and incubating sufficiently to ensure full conversion to product, as confirmed by HPLC (11).

### Equilibrium binding assays

Equilibrium binding of TDG to a G·T^F^ substrate analog, in the presence or absence of nucleobases (U, hmU or T), was analyzed using EMSAs, performed essentially as described (21). Note that T^F^ is the 2′-fluoroarabino analogue of dT (described above). Samples contained 10 nM G·T^F^ DNA, 5 nM to 1000 nM TDG and 10 mM of the nucleobase (where used). Samples were incubated at room temperature for 30 min, loaded onto a 6% native denaturing polyacrylamide gel and run for 60 min, 100 V at 4°C. Gels were imaged using a Typhoon 9400 variable mode imager (GE Healthcare).

## RESULTS

### New conditions for crystalizing DNA-bound TDG

The first structures of DNA-bound TDG were of E·S or E·P complexes, with the catalytic domain of human TDG (TDG^cat^) (22–24). Crystals were obtained under neutral pH conditions and the enzyme bound to the DNA with a stoichiometry of 2:1 (TDG^cat^:DNA), one subunit at the target site and a neighboring subunit bound to non-specific DNA (Condition I, Table 1). However, biochemical studies indicate that TDG binds DNA with a stoichiometry of 1:1 under limiting enzyme conditions and that a single TDG subunit possesses full catalytic activity (22,48,68). It seems likely that the 2:1 stoichiometry is attributable to the DNA construct used for crystallization, a 22-bp duplex with 3′-dA and 3′-dT overhangs that forms a long contiguous strand in the crystals. These conditions yielded crystals that were mostly of fairly poor quality, and many had to be screened to identify a few that diffracted with acceptable resolution (2.8–3.0 Å). A different approach for crystallizing DNA-bound TDG^cat^ was reported by Hashimoto *et al*., yielding structures with improved resolution, up to 2.49 Å (Condition II, Table 1) (25,69). While these conditions yield a binding stoichiometry of 1:1, which is likely more biologically relevant than 2:1 binding (22,48,68), the pH is 4.6, and TDG activity is not detected at pH values below ∼5.5 (70,71).

**Table 1.** Crystallization conditions used for obtaining structures of DNA-bound TDG^cat^

	Condition I	Condition II	Condition III
	(PDB ID: 2RBA)	(PDB ID: 4FNC)	(PDB ID: 4Z47)
Space group	P6_5_	C2	C2
Resolution (Å)	2.79	2.49	1.45
*R*_work_/*R*_free_	0.230/0.276	0.224/0.267	0.140/0.195
Wilson B-factor (Å^2^)	87.2	58.7	23.5
No. of atoms, protein and DNA	3847	2716	2767
No. of water molecules	0	38	281
Stoichiometry (TDG^cat^:DNA)	2:1	1:1	1:1
pH (mother liquor)	7.0	4.6	6.0
DNA construct	22 bp A/T overhang	28 bp T overhang	28 bp no overhang
R.m.s.d to new structure (Å)	0.40	0.27	

For each type of crystallization condition, the PDB ID is given for the highest-resolution structure reported to date. R.m.s.d. values were obtained from alignment of the protein using PyMOL; for the 2:1 complex (PDB ID: 2RBA), the TDG^cat^ subunit bound to the abasic analog was used for alignment.

**Figure 2.**
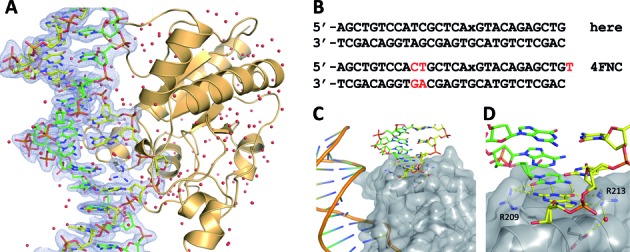
New conditions used here for generating high-resolution structures of DNA-bound TDG^cat^. (**A**) Structure of the enzyme–product (E·P) complex generated by incubating TDG^cat^ with DNA containing a G·U mispair, solved at 1.45 Å resolution (PDB ID: 4Z47). The target DNA strand is yellow, the complementary strand green, TDG^cat^ is in light orange and water molecules are red spheres. The 2*F*_o_-*F*_c_ omit map, contoured at 1.0 σ, is shown for the DNA and the Arg275 side chain of TDG^cat^. (**B**) DNA sequence used for the structures reported here (labeled ‘here’), and the sequence used for a structure of the E·P complex reported by Hashimoto *et al*. (25) (labeled ‘4FNC’). For both DNAs, ‘x’ represents the abasic site. (**C**) Interactions involving two Arg resides and a cavity on the TDG^cat^ surface with the terminal G·C base pair of a symmetry-related DNA molecule. The DNA shown in cartoon format is that to which TDG^cat^ is specifically bound (at the abasic site). (**D**) Close-up view of the symmetry-related DNA-TDG^cat^ contacts shown in panel C.

Seeking to obtain higher quality crystals and conditions that are more biologically relevant, we modified the approach of Hashimoto *et al*., by altering the DNA and screening for conditions at pH values above 4.6. We obtained high-quality crystals at pH 5.5 and 6.0, and optimized the conditions at pH 6.0, where TDG exhibits full glycosylase activity (70,71). We solved structures of E·P complexes resulting from TDG^cat^ action on DNA substrates including G·T, G·U, G·hmU, G·fC and G·caC. The resolution of these structures, up to 1.45 Å (Supplementary Table S1), is generally much better than that of any previously reported structure (Table 1). The new structures reveal hundreds of water molecules (Figure 2A, Table 1), far more than observed in previous structures of TDG^cat^; some mediate substrate binding and may function in catalysis (*vide infra*). As expected, these structures adopt a very similar overall fold relative to structures obtained from previous crystallization conditions (see RMSDs, Table 1).

We note that the 28 bp DNA used for these new structures differs slightly but significantly from that used by Hashimoto *et al*. (25,69), which had a 3′ dT overhang on the strand containing the target base, and a different sequence (Figure 2B). One or both of these differences, and/or the change in pH, may account for the improved resolution of our structures. Notably, the terminal dG of the target strand, and its dC partner in the complementary strand, are contacted by Arg213 and Arg209, respectively, from a symmetry-related molecule of TDG^cat^ (Figure 2C and D). These contacts are also seen in the structure reported by Hashimito *et al*. (25), but the interaction might be somewhat destabilized by the 3′-dT overhang (only the 5'-phosphate of the dT overhang is present in the electron density for their structure). There is also a symmetry-related contact involving the side chain of His179 and a DNA backbone phosphate; the dinucleotide sequence containing this phosphate differs for our DNA (TpC) relative to that used by Hashimoto *et al*. (CpT) (Figure 2B) (25). Notably, structures reported here exhibit two slightly different conformations for the dC nucleotide contacted by His179, and for its 3′ (dG) neighbor.

### The excised base dissociates from the product complex

**Figure 3.**
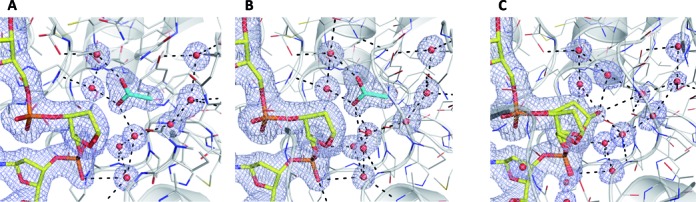
New structures demonstrate that the excised base is absent from enzyme–product complexes resulting from TDG^cat^ action on various substrates. (**A**) Close-up view of the active site for the enzyme–product (E·P) complex resulting from TDG^cat^ action on a G·hmU substrate, solved at 1.72 Å (PDB ID: 4XEG). The abasic sugar is flipped into the active site, with C1’-OH pointing toward the viewer. The excised base is clearly absent. Coloring is by element, with carbon atoms of the DNA in yellow, the enzyme in white and the acetate in cyan (O, N and P atoms are red, blue, and orange, respectively). Red spheres are water molecules. The 2*F*_o_-*F*_c_ omit map, contoured at 1.0 σ, is light blue. The same coloring scheme is used for the two other panels. (**B**) Structure of the E·P complex for TDG^cat^ acting on a G·U mispair, solved at 1.45 Å (PDBID: 4Z47). Uracil was present at a concentration of 10 mM in solutions used for sample preparation and all crystallization steps. (**C**) Structure of the E·P complex generated from TDG^cat^ action on a G·U mispair, solved at 1.75 Å (PDB ID: 4Z3A), obtained from crystals grown in acetate-free conditions.

To address the proposal that TDG traps the excised hmU base in the product complex, we consider our new structure of this complex, solved at 1.72 Å resolution (Figure 3A). The electron density is excellent and clearly defines the constituents of the active site, which include the flipped abasic nucleotide, as expected, and many water molecules, most not detected in previous structures. Importantly, the electron density demonstrates unambiguously that an acetate molecule, rather than the excised hmU base, resides in the TDG^cat^ active site. Thus, hmU has dissociated from the product complex. The presence of acetate in the active site is likely explained by its interaction with three ordered water molecules and its high concentration (300 mM) in the mother liquor used for crystallization. Notably, the same acetate concentration was present in the mother liquor used by Hashimoto *et al*. for their structure of the E·P complex that was reported to contain the excised hmU base (PDB ID: 4FNC) (25). The electron density for PDB entry 4FNC, as reported by the electron density server (EDS) (72) and PDB_REDO (73) validation servers, is compatible with acetate and neighboring water molecules, though given the lower resolution (2.^49^ Å), interpretation may not be straightforward.

Given that hmU is clearly absent from the enzyme–product complex, we also determined the structure of product complexes resulting from the action of TDG^cat^ on other substrates. Structures of E·P complexes generated from G·fC and G·caC substrates, solved at 2.02 Å and 2.45 Å resolution, respectively, give the same result (Supplementary Table S1. The active site contains the abasic sugar, several water molecules and an acetate molecule, but not the excised base (not shown).

We also investigated whether the nucleobase, if present at high concentration, might occupy the product complex. We crystallized E·P complexes that were generated by incubating TDG^cat^ with a G·T, G·U, or G·hmU substrate in buffer that also contained a 10 mM concentration of the relevant nucleobase (T, U or hmU). Importantly, the same concentration of the nucleobase was also present in the mother liquor for crystallization and the solution used to cryoprotect the crystals. The resulting structures reveal that the nucleobase is not bound in the product complex, despite its high concentration during sample preparation, crystallization and cryoprotection. This is clearly demonstrated by the E·P complex generated from a G·U substrate (Figure 3B), solved at 1.45 Å resolution (Supplementary Table S1). The active site contains an acetate molecule, the abasic sugar and water molecules, but not the excised uracil base. Likewise, a 1.83 Å structure of the E·P complex generated from a G·T substrate (Supplementary Table S1) shows that thymine is absent from the active site, despite the presence of 10 mM thymine at all stages of crystal production (not shown). We note that the 10 mM concentration of the nucleobases used here for TDG is much higher than that used for structures of glycosylases that do bind their cognizant nucleobase in the E·P complex, including TAG, UNG and SMUG1 (28,29,32,33). Thus, TDG has negligible affinity for the excised base in the product complex.

Notably, all of the structures described above were generated from crystals that were grown using mother liquor that contained 300 mM acetate. To rule out the possibility that acetate displaces the excised base in the product complex, we grew crystals of a TDG^cat^ product complex, generated from a G·U substrate, using mother liquor that did not contain acetate and solved a structure at 1.75 Å resolution (Supplementary Table S1). The electron density demonstrates that the active site does not contain acetate or the excised uracil base (Figure 3C). Rather, it contains only water molecules and the abasic sugar.

### NMR studies indicate that the excised base dissociates from the product complex in solution

We also sought to determine whether the excised base is trapped in the TDG^cat^ product complex in aqueous solution. Given the exquisite sensitivity of backbone amide ^1^H-^15^N resonances to structural and environmental changes, NMR chemical-shift-perturbation experiments are a powerful and widely used method to monitor protein–ligand interactions (74–76). Our approach was to compare the backbone ^1^H-^15^N chemical shifts of TDG^cat^ in two different samples. The first sample included TDG^cat^ with DNA containing a pre-existing abasic site (G·AP); the second sample was generated by incubating TDG^cat^ with DNA substrate to give complete conversion to product (confirmed by HPLC). If the excised base dissociates from the product complex then there should be no substantial chemical shift differences because the resulting complexes would be identical (TDG^cat^ bound to G·AP DNA). However, if the excised base is trapped in a ternary product complex and forms multiple interactions with active-site groups, as suggested (25), then substantial and numerous chemical shift perturbations should be observed relative to the binary complex of TDG^cat^ bound to abasic DNA.

**Figure 4.**
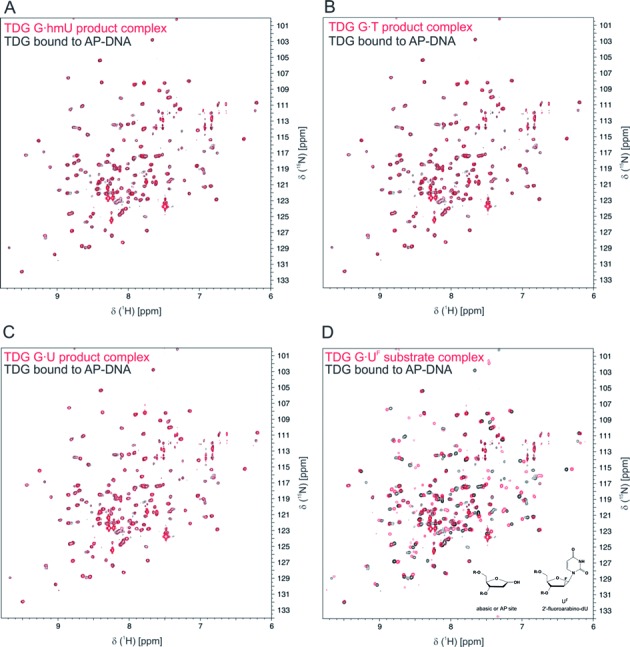
NMR studies also indicate that the excised base is released from enzyme–product complexes. All four panels shown an identical ^15^N-TROSY spectrum for TDG^cat^ bound to AP-DNA (black peaks); the sample was prepared by adding TDG^cat^ to purified abasic DNA (AP-DNA). The red peaks in panels **A**–**C** are ^15^N-TROSY data for enzyme–product complexes resulting from TDG^cat^ action on various substrates, including G·hmU (A), G·T (B) and G·U (C). The absence of substantial chemical shift changes indicates that the excised base is released from the enzyme–product complex. (**D**) The red peaks are ^15^N-TROSY data for TDG_cat_ bound to DNA containing a G·U^F^ base pair; U^F^ is a dU analog that flips into the active site but cannot be cleaved by TDG. Substantial chemical shift changes are observed for most backbone ^15^N-^1^H resonances; the DNA differs at only one nucleotide (AP site versus U^F^, see inset). Samples contained 0.25 mM ^15^N-labeled TDG^cat^, 0.30 mM DNA, 5 mM Tris–HCl pH 7.5, 0.2 M NaCl, 0.2 mM DTT, 0.2 mM EDTA, 10% D_2_O.

To obtain the baseline NMR spectrum, we collected a ^15^N-TROSY experiment for TDG^cat^ bound to abasic DNA (Figure 4A, black peaks). We collected the same data for a product complex generated by incubating TDG^cat^ with otherwise identical DNA that contained a G·hmU mispair (Figure 4A, red peaks). These two NMR spectra exhibit no substantial differences in backbone ^1^H-^15^N chemical shifts, indicating that the excised hmU base dissociates from the product complex. The same NMR experiments were collected for product complexes generated by incubating TDG^cat^ with DNA containing a G·T mispair (Figure 4B, red peaks) or a G·U mispair (Figure 4C, red peaks). These spectra also exhibit no substantial differences relative to the baseline spectra of TDG^cat^ bound to DNA containing a pre-existing abasic site (Figure 4B and C, black peaks), indicating that the excised base dissociates from the product complex generated by TDG^cat^ action on a G·T or a G·U substrate. Thus, the NMR results are fully consistent with our crystallographic findings.

To illustrate the potential extent of chemical shift changes that might be observed if the excised base was retained in the product complex, we collected a TROSY spectrum for TDG^cat^ bound to the identical DNA construct containing a G**·**U^F^ mispair; U^F^ is an analog of deoxyuridine that flips into the active site but is not excised by TDG (23,48,51,52). We find substantial chemical shift changes for dozens of backbone ^1^H-^15^N resonances of TDG^cat^ bound to G·U^F^ DNA (Figure 4D, red peaks) versus G·AP DNA (Figure 4D, black peaks). The predominant difference between these DNAs is the presence of uracil for G**·**U^F^ but not G·AP DNA (Figure 4D). Two other differences that may account for some chemical shift changes are the hydroxyl at C1′ for the abasic nucleotide, and the 2′-fluoro of U^F^. Nevertheless, it is likely that at least some of the extensive shift perturbations are due to the presence of the uracil base in G**·**U^F^ but not G**·**AP DNA, indicating that if the excised base were retained in the product complex then chemical shift changes would be expected in Figures 4A–C.

### DNA-free TDG also exhibits negligible affinity for nucleobases that it excises from DNA

Given that that the nucleobases excised by TDG do not bind with significant affinity to the enzyme–product complex, we investigated their potential binding to DNA-free TDG. We performed NMR experiments for TDG^cat^ in the absence and presence of uracil, thymine, or hmU at a concentration of 10 mM (Supplementary Figure S1). Remarkably, the NMR spectra reveal no significant differences, indicating that TDG^cat^ has very weak affinity for these nucleobases, *K*_d_ >> 10 mM, even tough it readily excises them from DNA. We also investigated the potential interaction of these bases with full-length TDG, by examining their effect on TDG binding to DNA containing a G·T^F^ mispair, where T^F^ is an analog of deoxythymidine that flips into the active site but is not excised by TDG (23,48,51,52) (Figure 4D shows U^F^). Binding to G·T mispairs is weak relative to other substrates and is more sensitive to agents that perturb substrate binding (23,24,48,51,70). TDG binding to G·T^F^ DNA is not detectably altered by the presence of uracil, thymine or hmU at high (10 mM) concentration (Supplementary Figure S2). Together, the results indicate that free TDG has negligible affinity for U, T or hmU.

### Solvent-filled channel to the enzyme active site

**Figure 5.**
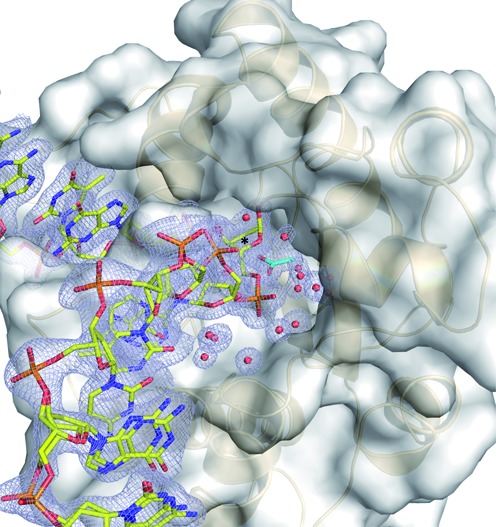
The new structures reveal a solvent-filled channel to the active site for DNA-bound TDG. The structure of the enzyme–product complex resulting from TDG^cat^ action on a G·U DNA substrate (PDB ID: 4Z47, 1.45 Å) reveals a solvent-filled channel from the active site to the enzyme surface that runs along the target DNA strand. TDG^cat^ is shown in both space-filling and cartoon modes, the DNA is in stick format, with the target strand colored yellow (complementary strand not shown for clarity). Water molecules are shown as red spheres, and the acetate is cyan. The 2*F*_o_-*F*_c_ omit map, contoured at 1.0 σ, is shown light blue for the target DNA strand, acetate and water molecules.

The high-resolution structures reported here, which reveal hundreds of water molecules, indicate that the active site of DNA-bound enzyme is accessible to solvent. Specifically, the structures reveal a network of water molecules extending from the active site down along the target strand of the DNA toward the 5′ end (Figure 5). It seems reasonable that this channel could provide an escape route for the excised base, though this may require movement of the enzyme or DNA (or both). The channel may also be important for catalysis, given the previous finding that TDG excision of caC is acid catalyzed, involving a proton derived from solvent rather than a general acid of the enzyme (71). The solvent-filled channel revealed here for product complexes suggests a mechanism by which the caC base can be protonated (via solvent) in the enzyme–substrate complex.

### Anomeric form of the abasic sugar in product complexes

**Figure 6.**
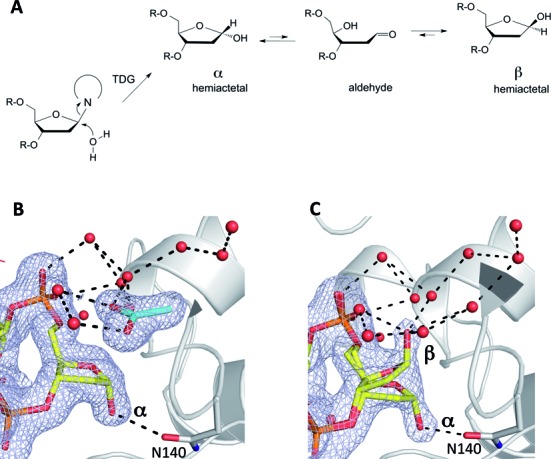
Anomeric structure of the abasic sugar observed in TDG enzyme-product complexes. (**A**) Abasic sites exist in several potential forms for enzyme-free DNA in solution. The α and β anomers of the cyclic hemiacetal predominate (together comprising 99%); minor forms are the ring-opened aldehyde and a hydrated aldehyde (not shown). For the TDG reaction, the initial enzyme-bound product resulting from C-N bond cleavage is expected to be the α anomer. (**B**) The α anomer is the predominant form of the abasic sugar observed for all TDG^cat^ product complexes that include an acetate molecule in the active site (crystallized in the presence of 0.3 M acetate). (**C**) A roughly equal mix of α and β anomers is observed in a TDG^cat^ product complex that lacks acetate in the active site. The β anomer appears to be stabilized by hydrogen bonds from C1’-OH to water molecules in the active site pocket.

Interestingly, our new structures indicate that absence of the excised base (or acetate) in the active site impacts the equilibrium between the two potential anomeric forms of the abasic sugar produced by TDG. Abasic sites adopt several potential forms in free DNA (Figure 6A), including a cyclic hemiacetal, a ring-opened aldehyde and a hydrated aldehyde (latter not shown). The cyclic hemiacetal predominates (99%), existing as a mix of α and β anomers that interconvert through the aldehyde (77–79). The α anomer is the initial form produced by TDG and other monofunctional DNA glycosylases that use water as the nucleophile (Figure 6A) (23,44). For product complexes crystallized in the presence of acetate, the α anomer clearly predominates (Figure 6B). However, for those crystallized under acetate-free conditions, the abasic sugar exists in a roughly equal mix of α and β anomers, each forming different interactions involving C1’-OH (Figure 6C). The β anomer contacts several ordered water molecules in the active-site, while the α anomer contacts the O_δ_ of Asn140 (Figure 6C), the side chain that coordinates the putative water nucleophile in the E·S complex (23). Interestingly, refinement indicates that a water molecule may occupy the site vacated by C1’-OH of the α but not the β anomer (not shown). This water molecule would interact with Asn140-O_δ_, the backbone O of Thr197, and O4’ of the abasic sugar, some of which bind the putative water nucleophile in the E·S complex (23). Our findings show that dissociation of the excised base is required for TDG binding to the β anomer of the abasic sugar. They also raise the possibility that the abasic sugar equilibrates between the α and β anomers in the TDG active site, and that this equilibrium is strongly shifted towards α by the presence of acetate.

## DISCUSSION

### Improved crystallization conditions for TDG

Our new conditions for crystallizing DNA-bound TDG^cat^ yield structures of unprecedented resolution for this enzyme, maintain the 1:1 binding stoichiometry observed by Hashimoto *et al*. (25,69), and are at pH 6.0, where TDG is fully active (70,71). The conditions yield crystals in a matter of days and only a few are typically needed to obtain high-quality data. As such, the approach may allow the determination of yet unsolved structures of DNA-bound TDG, and should make it feasible to obtain structures that could be mechanistically informative, i.e., of an active-site mutation, but which might not have been pursued using previous conditions, due to unpredictable crystal quality or moderate resolution. The new structures reveal hundreds of water molecules, some mediating enzyme–substrate interactions. For example, water molecules bridge enzyme contacts with the 5′-phosphate of the abasic nucleotide and the two phosphates 3′ of the abasic site (not shown), and a network of water molecules populate the active site. The structures reveal a solvent-filled channel to the active site (Figure 5), providing a potential pathway for proton transfer from bulk solution, which may be needed for acid catalysis of caC excision given that no enzyme group appears to perform general acid catalysis (71). Notably, most of the water-mediated enzyme–DNA contacts observed in our new structures were not detected in previous structures of DNA-bound TDG. Indeed, except for the putative water nucleophile, no water molecules were observed in the four structures obtained using the initial crystallization conditions (Type I, Table 1) (22–24).

### The excised base dissociates from the product complex

Our structures demonstrate that the excised base is not retained in the enzyme–product complex, regardless of the substrate from which it is derived (G·hmU, G·U, G·T, G·fC or G·caC). These crystallographic findings are confirmed in solution by NMR, for product complexes generated from G·hmU, G·U and G·T substrates. Moreover, the same result is obtained even when the relevant base is supplemented to a concentration of 10 mM during sample preparation, crystal growth and cryoprotection. Our results are consistent with previous findings that the excised base (uracil) is not observed in a structure (2.35 Å) of the enzyme–product complex derived from a G·U substrate for MUG from *E. coli* (27), the most closely related glycosylase to TDG (32% identity) (22). Our structures also demonstrate that acetate resides in the active site of the E·P complex for crystals grown in the presence of acetate (300 mM). Moreover, crystals grown under acetate-free conditions yield structures of the E·P complex that contain only water molecules in the active site. Our findings conflict with a previous report that the excised hmU base remains trapped in the TDG^cat^ product complex generated from a G·hmU substrate (25). The crystallization conditions used in the previous studies also included acetate (300 mM), and are very similar to those used here except for differences in pH and the DNA construct (Table 1, Figure 2). The high-resolution (up to 1.45 Å) structures here suggest that acetate and water molecules account for the electron density that had been attributed to the excised hmU base in the previous structure (PDB ID: 4FNC), solved at 2.49 Å resolution.

### How does the excised base depart the product complex?

Observation that the excised base is absent from the product complex raises the question of how it is released. One plausible mechanism is that the excised base escapes through the solvent-filled channel revealed by our new structures, which could involve movement of the enzyme or DNA (or both). Notably, MUG has a similar though seemingly smaller channel, and it releases the excised uracil base from an E·P complex (27). By contrast, UNG, which traps the excised uracil in its product complex, lacks the channel found in TDG and MUG (29,34). UNG also forms a strong hydrogen bond with the excised uracil (30,31), which likely helps to retain it in the E·P complex. Alternatively, it is possible that TDG releases the excised base through a different region of the active site, but this would require substantial structural changes given that no other obvious escape route is evident (22,25). Another possibility is that release of the excised base depends on partial or full dissociation of abasic DNA, as we previously suggested (80). However, our new findings indicate that dissociation of the excised base might precede that of abasic DNA, by escaping through the channel. Additional studies are needed to establish the mechanism of base release from TDG. It will also be of interest to determine the kinetic parameters for dissociation of the excised base and abasic DNA.

### TDG has negligible affinity for isolated nucleobases

Our findings reveal that TDG has no significant affinity for isolated nucleobases, even though it binds specifically to DNA containing these bases and readily excises them from DNA. TDG does not bind uracil, thymine or hmU, even at a 10 mM concentration, indicating very low affinity (*K*_d_ >> 10 mM). Likewise, enzyme–product complexes also lack significant affinity for free nucleobases. These results are consistent with previous findings that TDG is not inhibited by thymine, uracil, 5-fluorouracil and 3,*N*^4^-ethenocytosine (ϵC), at concentrations of 5 mM (80,81). Unlike TDG, many DNA glycosylases exhibit substantial affinity for free nucleobases. Fellow superfamily member UNG binds uracil with a *K*_d_ of about 0.1 mM (at pH 7.5) (30,82) and TAG binds m^3^A with a *K*_d_ of 0.04 mM (35). Our findings for TDG are also remarkable given that it exhibits high specificity for binding to DNA that contains the substrate base pairs examined here. For example, binding is nearly 500-fold tighter for DNA containing a G·U mispair (*K*_d_ = 0.6 nM) relative to non-specific DNA (*K*_d_ = ∼0.3 uM) (48). Studies of TDG^cat^ indicate that, relative to G·U, binding is 4- and 10-fold tighter to G·fC and G·hmU, respectively (24). While some of this specificity is probably attributable to base-pairing properties, much is undoubtedly due to interactions with the flipped target base. Previous structures of E·S complexes (G·U, G·caC or A·caC) show that TDG forms contacts with the flipped base at positions including O2, N3, O4 or N4H_2_, and the carboxyl oxygen of caC (23,24), suggesting similar contacts for other substrates (hmU, T, 5-halo-U, fC). These contacts seem likely to help retain the flipped base in the E·S complex and/or stabilize the anionic form of the base that serves as the leaving group in the chemical step of the reaction. Yet, despite these contacts, TDG possesses no significant affinity for substrate bases that are isolated from DNA.

### Dissociation of the excised base might stabilize the enzyme–product complex with abasic DNA

Our results, together with previous findings for other glycosylases, indicate that release of the excised base impacts the anomeric form adopted by the abasic sugar, which could potentially strengthen the product complex. Findings here indicate that the anomeric form of the flipped abasic sugar depends on whether the E·P complex is occupied with acetate. Structures with acetate exhibit only the α conformation, while those lacking acetate exhibit a roughly equal mix of α and β anomers. This is potentially relevant because the β anomer forms more contacts with TDG (including water mediated ones) than the α anomer (Figure 6), suggesting that TDG might bind tighter to the β versus α anomer. In support of this idea, the abasic sugar adopts only the β anomer in new structures of E·P complexes obtained using a construct of TDG that contains additional N-terminal residues relative to TDG^cat^ (Malik, S.S., Coey, C.T., Pozharski, E. *et al*. manuscript in preparation). Our new structures here indicate that release of the excised base (and the absence of acetate) is essential for adoption of the β anomer, and this idea is supported by structures of other DNA glycosylases. A structure of the MUG product complex (2.35 Å) shows that the excised uracil is absent and the abasic sugar adopts the β anomer (27). Structures of E·P complexes for UNG show that the anomeric form of the abasic sugar depends on whether the excised base remains in the active site. Two structures that retain the excised uracil feature only the α-anomer (29,34), while the β anomer predominates in a structure for which the excised uracil base has dissociated (L272A-UNG variant) (29). Taken together, these observations raise the possibility that release of the excised base could potentially confer tighter binding of TDG to its abasic DNA product, by allowing formation of the β anomer.

## ACCESSION NUMBERS

Coordinates and structure factors have been deposited in the Protein Data Bank (http://www.rcsb.org/) with accession numbers 4XEG, 4Z7B, 4Z7Z, 4Z47, 4Z3A and 5CYS.

## Supplementary Material

SUPPLEMENTARY DATA
