# Supplementary material for: Thymine DNA glycosylase exhibits negligible affinity for nucleobases that it removes from DNA
Source: Nucleic Acids Res. 2015 Sep 10;43(19):9541–52. doi: 10.1093/nar/gkv890 (PMC4627079; doi:10.1093/nar/gkv890)
Supplement: SUPPLEMENTARY DATA [file supp_43_19_9541__index.html]

Thymine DNA glycosylase exhibits negligible affinity for nucleobases that it removes from DNA — SUPPLEMENTARY DATA 

# Thymine DNA glycosylase exhibits negligible affinity for nucleobases that it removes from DNA

## SUPPLEMENTARY DATA

- SUPPLEMENTARY DATA
