# Supplementary material for: Thymine DNA glycosylase exhibits negligible affinity for nucleobases that it removes from DNA
Source: Nucleic Acids Res. 2015 Sep 10;43(19):9541–52. doi: 10.1093/nar/gkv890 (PMC4627079; doi:10.1093/nar/gkv890)
Supplement: SUPPLEMENTARY DATA [file supp_gkv890_nar-01113-f-2015-File008.pdf]

Supplementary Data for:

Thymine DNA glycosylase exhibits negligible affinity for nucleobases that it removes from DNA

Shuja S. Malik[1], Christopher T. Coey[1], Kristen M. Varney[1,2,3], Edwin Pozharski[1,2,3,*], and Alexander C. Drohat[1,2,*]

[1] Department of Biochemistry and Molecular Biology, University of Maryland School of Medicine, Baltimore, MD 21201, USA

[2] University of Maryland Marlene and Stewart Greenebaum Cancer Center, Baltimore, MD 21201, USA

[3] Center for Biomolecular Therapeutics, Institute for Bioscience and Biotechnology Research, Rockville, MD 20850, USA

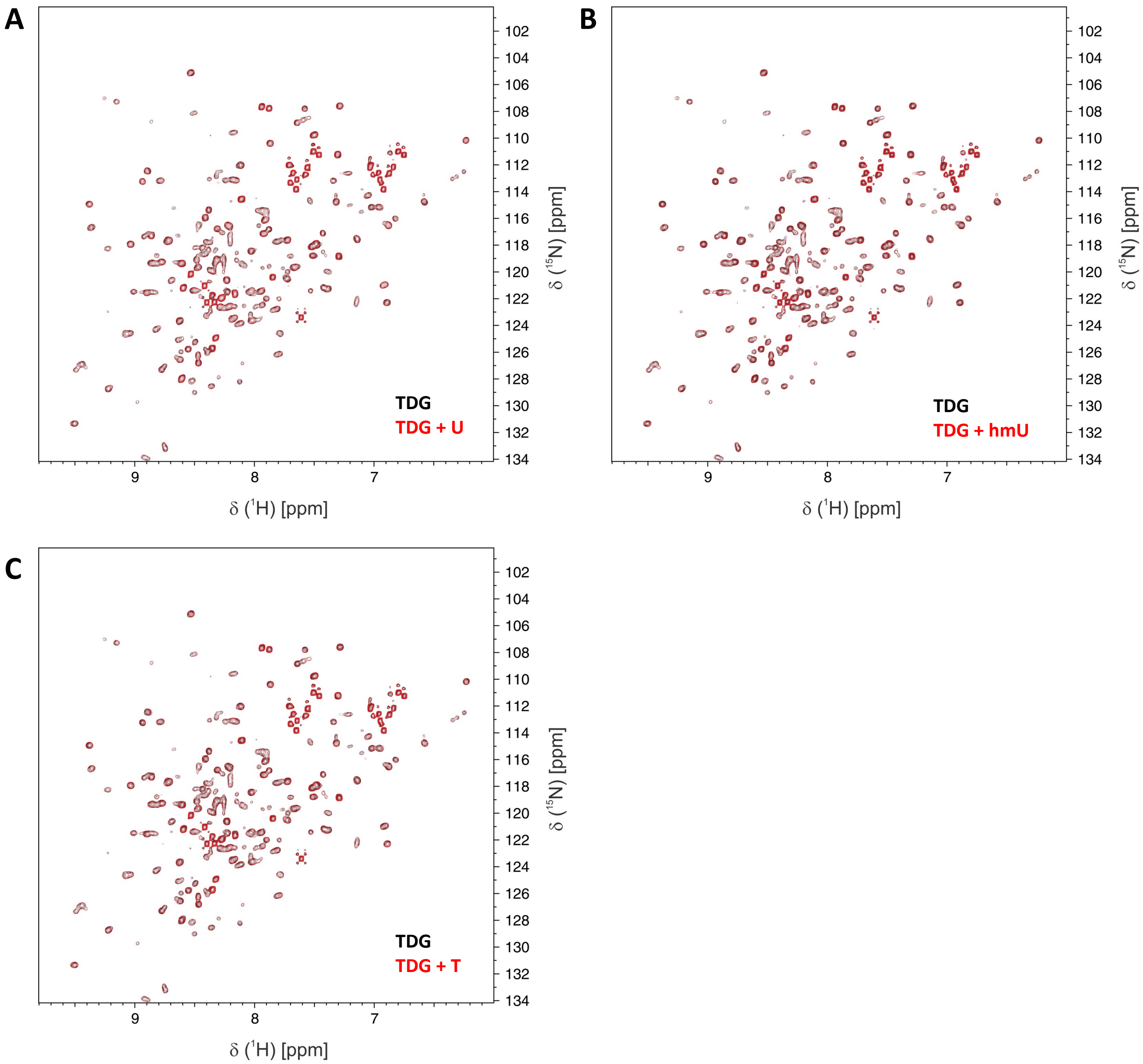

Figure S1. TDG^{cat} exhibits no appreciable binding to uracil, thymine, or hmU. All three panels shown an identical ^{15}N-HSQC spectrum for free TDG^{cat} (black peaks). The red peaks in panels A-C are ^{15}N-HSQC spectra for TDG^{cat} together with a 10 mM concentration of uracil (**A**), thymine (**B**), or hmU (**C**). No substantial chemical shift differences are observed in any of the three panels, indicating that TDG^{cat} does not bind detectably to any of the three bases. Samples contained 0.54 mM ^{15}N-labeled TDG^{cat}, 20 mM sodium phosphate pH 6.5, 0.15 M NaCl, 0.2 mM DTT, 0.2 mM EDTA, 7% D_2O.

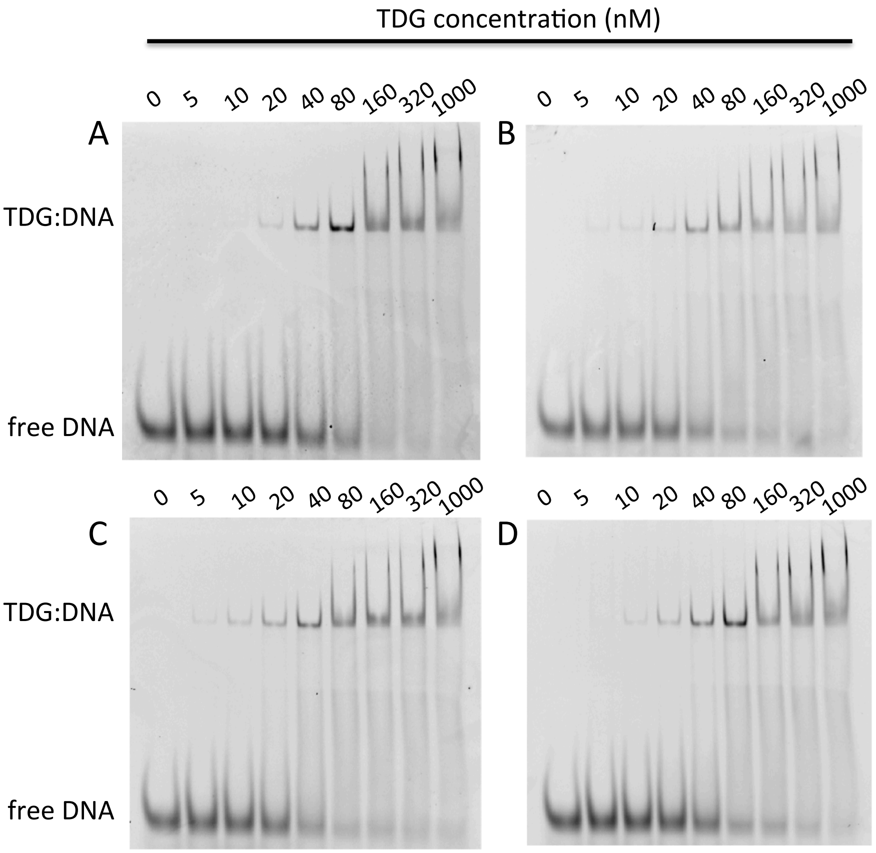

Figure S2. Binding of TDG to DNA containing a G·T^F analog is not altered by nucleobases. Electrophoretic mobility shift assays (EMSAs) were performed for TDG (full length) binding to a 28 bp DNA containing a G·T^F mispair, where T^F is 2'-fluoroarabino-dT, a dT analog that flips into the active site but is not excised by TDG. The four panels show EMSAs performed under identical conditions for TDG binding to G·T^F DNA in the absence of a nucleobase (**A**) and in the presence of 10 mM concentration of (**B**) uracil, (**C**) thymine, and (**D**) hmU. The concentration of DNA (6-FAM labeled) was 10 nM, and the TDG concentration was varied as shown at the top of the gels. The results suggest a K_d of roughly 40 nM and indicate that the nucleobases do not alter TDG binding to G·T^F DNA.

Supplementary Table S1. Data collection and refinement statistics

	EP from GhmU (PDBID: 4XEG)	EP from GfC (PDBID: 4Z7B)	EP from GT, (PDBID: 4Z7Z)	EP from GU, excess U (PDBID: 4Z47)	EP; GU, no acetate (PDBID: 4Z3A)	EP from GcaC (PDBID: 5CYS)
**Data collection**						
Space Group	C2	C2	C2	C2	C2	C2
Cell Dimensions						
a, b, c (Å)	89.2, 53.4, 82.2	96.2, 53.8, 81.2	90.7, 53.5, 82.0	89.3, 53.3, 82.3	95.2, 53.6, 81.4	97.9, 53.8, 81.2
β (°)	95.4	95.6	95.5	95.3	95.1	96.1
Resolution (Å)	39-1.72 (1.75-1.72)	33-2.02 (2.07-2.02)	39-1.83 (1.87-1.83)	46-1.45 (1.47-1.45)	32-1.72 (1.76-1.72)	33-2.45 (2.55-2.45)
R_{pim}	0.127	0.055	0.055	0.027	0.059	0.113
Mean $I/\sigma I$	23.8 (1.4)	10.1 (0.7)	10.2 (0.4)	15.9 (1.2)	6.6 (0.6)	5.2 (0.6)
$CC_{1/2}$	0.997 (0.5)	0.998 (0.36)	0.992 (0.3)	0.999 (0.65)	0.997 (0.3)	0.992 (0.34)
Completeness (%)	97.2 (62.1)	98.8 (95.4)	98.5 (91.5)	94.9 (89.8)	97.7 (92.4)	99.5 (96.9)
Redundancy	6.6 (3.8)	5.0 (4.8)	8.8 (3.0)	11.7 (11.0)	8.8 (4.6)	4.9 (4.7)
Wilson B-factor ($Å^2$)	29.8	32.3	34.7	23.5	23.1	49.4
**Refinement**						
Program	REFMAC5	BUSTER-TNT	BUSTER-TNT	REFMAC5	BUSTER-TNT	BUSTER-TNT
Resolution (Å)	39-1.72	32-2.02	39.3-1.83	46-1.45	32-1.72	33-2.45
No. of reflections	38.012	26,944	34,036	61,716	42,189	15,396
R_{work}/R_{free}	0.188/0.232	0.187/0.233	0.193/0.237	0.140/0.195	0.199/0.226	0.165/0.227
Number of atoms						
Protein	1590	1530	1560	1591	1571	1558
DNA	1176	1135	1176	1176	1188	1176
Water	304	144	209	281	185	210
Other	20	12	8	16		4
B-factors ($Å^2$)						
Protein	34.6	46.2	49.0	33.8	43.1	65.0
DNA	45.4	72.5	65.3	43.6	58.4	93.1
Water	47.2	54.4	53.6	46.8	50.4	51.6
Acetate	32.9	47.6	40.1	29.8	-	57.0
Ramachandran Plot						
Favoured (%)	96	97.5	98	98	98	95
Allowed (%)	2	2	2	2	2	4.5
Outliers (%)	2	0.5	0	0	0	0.5
RMSD from ideal						
Bond lengths (Å)	0.017	0.010	0.010	0.023	0.010	0.010
Bond angles (°)	1.90	1.01	0.99	2.30	0.99	1.04

Values shown in parenthesis are for highest resolution shell. The Ramachandran analysis was performed using Molprobity [1]. Wilson B-factor estimated by phenix.xtriage. Number of atoms includes all atom records explicitly included in the model, including alternate positions.

1. Chen, V.B., W.B. Arendall, III, J.J. Headd, D.A. Keedy, R.M. Immormino, G.J. Kapral, L.W. Murray, J.S. Richardson, and D.C. Richardson, *MolProbity: all-atom structure validation for macromolecular crystallography.* Acta Crystallographica Section D, 2010. **66**(1): p. 12-21.
